# Harnessing brain–body communication to understand cancer

**DOI:** 10.1101/gad.352292.124

**Published:** 2024

**Authors:** Erica K. Sloan

**Affiliations:** Monash Institute of Pharmaceutical Sciences, Monash University, Melbourne, Victoria 3052, Australia

**Keywords:** brain–body, physiology, symposium

## Abstract

Solid tumors that arise in the body interact with neurons, which influences cancer progression and treatment response. Here, we discuss key questions in the field, including defining the nature of interactions between tumors and neural circuits and defining how neural signals shape the tumor microenvironment. This information will allow us to optimally target neural signaling to improve outcomes for cancer patients.

## Abstract (precis)

In this Outlook, Sloan addresses the open questions in our current understanding of the interactions between solid tumors and host neural circuits and how they shape the tumor microenvironment.

Neural circuits linking the brain and body control much of our biology, influencing how and when we eat, how we maintain body temperature, and how we sense our surroundings. We are just beginning to appreciate how the emerging cancer neuroscience field can leverage this understanding of brain–body neural circuits to gain insights into cancer pathophysiology that can improve health. The 88th Cold Spring Harbor Laboratory Symposium on Brain Body Physiology highlighted the neuroscience knowledge and tools that may be used to expand our understanding of the role of neural signaling in tumor biology.

Early histological documentation of nerve fibers within cancers has been given clinical relevance by recent studies that suggest that neural innervation is associated with worse outcomes for patients (Ferdoushi et al. 2021; Li et al. 2022). Now, a growing body of preclinical and clinical studies shows that modulating the peripheral nervous system influences the trajectory of cancer by impacting the growth and spread of solid tumors through the body (Thaker et al. 2006; Sloan et al. 2010; Le et al. 2016) and the response of the cancer to treatment (Chang et al. 2023). These observations complement well-studied interactions between the central nervous system and tumors that arise in the brain.

## Why would a tumor in the body benefit from connecting with the peripheral nervous system?

Seen from the perspective of the tumor, tapping into the neural network may provide significant advantages. Efferent neural signaling gives the tumor access to real-time information about the state of the body, which constitutes its macroenvironment. To respond to these signals, tumor cells express receptors for neurotransmitters from the sympathetic and parasympathetic nervous systems (Sloan et al. 2010). Connecting with afferent sensory innervation, which is activated by mechanical or chemical stimuli, may allow the tumor to communicate with the brain. In addition, antidromic neuropeptide signaling from sensory nerve terminals within the tumor has been shown to shape the immune profile of the tumor itself (Balood et al. 2022; McIlvried et al. 2022).These hardwired connections allow rapid communication that complements the soluble forms of communication that have been the focus of cancer research for many decades.

The 88th Cold Spring Harbor Laboratory Symposium on Brain Body Physiology showcased the abundance of neuroscience knowledge and tools that may be leveraged to expand our fundamental understanding of tumor biology. Many key questions remain to be answered.

## What is the nature of interactions between solid tumors and neural circuits?

The cancer neuroscience field is just beginning to understand how tumors in the body interact with neurons. The types of neurons present in solid tumors will likely be influenced by the efferent and afferent neurons present in the host organ in which the tumor develops. Consistent with this idea, a recent analysis of patient samples found that the majority of nerve fibers in breast cancers stain positive for tyrosine hydroxylase, suggesting dominant sympathetic innervation that will activate catecholaminergic signaling within the tumor (Li et al. 2022). Mammary tissue likely also contains sensory and parasympathetic innervation, though it is currently unknown whether these neural subtypes are recruited into tumors. To accelerate research in this space, it will be important to define the type and extent of innervation across different tumor types, focusing on clinical samples rather than mouse models of cancer. In addition, it will be necessary to use methodology that can discriminate between neural subtypes, expanding beyond use of pan-neuronal markers. Use of single markers also may not be sufficient to define distinct neural populations; for example, tyrosine hydroxylase is expressed by sympathetic neurons and also by a subset of sensory neurons.

It will also be important to define the variability in the extent to which tumors engage with neural pathways in the body. Although the statement “tumors are densely innervated” is found in publications with remarkable frequency, evidence to support the magnitude of this claim is scarce. Clinical evaluations demonstrated the presence of nerve fibers and bundles in a subset of primary tumors (for example, 10% of pancreatic cancers and 30%–85% of breast cancers) (Ferdoushi et al. 2021; Li et al. 2022). However, the majority of these tumors are described as having “low nerve count” (Li et al. 2022), and some studies have relied on tissue microarrayed samples rather than full-face tissue sections (Ferdoushi et al. 2021), which may reduce the accuracy of the findings.

There is an opportunity to apply neuroscience tools for mapping and modulating the activity of neural circuits to mouse models of cancer to complement clinical observations of neural–tumor interactions. At the Symposium, Dr Jeremy Borniger discussed how orthotopically injected mammary tumors can distally alter the activity of neurons in the hypothalamus that regulate glucocorticoid circadian rhythms (Francis and Borniger 2021). This involved applying viral tract tracing and chemogenetic methods, approaches that have been relegated to the periphery of preclinical oncology until very recently. Given tissue-specific patterns of innervation, it is important that orthotopic cancer models are used in preclinical studies. When combined with tissue-clearing methodology and advanced microscopy, neural circuit mapping will allow us to define how manipulating the cancer (for example, through treatment) may impact tumor neural networks. Selective manipulation of specific neural types using chemogenetic and optogenetic tools will allow the impact of neural activity on progression of cancer to be investigated.

## How do neural signals shape tumors?

Research to understand how neural signaling modulates cancer progression has focused on the sympathetic nervous system (SNS), spurred on by a desire to understand how the “fight or flight” stress response accelerates metastatic spread (Thaker et al. 2006; Sloan et al. 2010). Mechanistic studies found that SNS signaling drives tumor cell invasion through cytoskeletal changes and increased production of matrix-degrading proteases. In addition to effects on tumor cells, SNS signaling shapes the tumor microenvironment. SNS activation remodels tumor vasculature, which provides pathways of tumor cell dissemination (Thaker et al. 2006; Sloan et al. 2010; Le et al. 2016) and affects innate and adaptive immune cells by modulating their function and recruitment to tumors (Sloan et al. 2010; Qiao et al. 2021). At the Symposium, Dr Elizabeth Repasky discussed the role of SNS signaling as a driver of immunosuppression through T-cell exhaustion and increased survival and frequency of myeloid-derived suppressor cells in the tumors. It will be important to characterize SNS regulation of other tumor components, including fibroblasts and stroma. Less is known about the impact of parasympathetic and sensory nerve fibers on the tumor microenvironment. However, recent findings that sensory nerves affect the functional status and recruitment of immune cells to melanoma and head and neck cancers suggest that it will be important to understand the net effect of different neural subpopulations on the tumor microenvironment (Balood et al. 2022; McIlvried et al. 2022).

## How can neural signals be targeted to improve patient outcomes?

Ultimately, the goal of cancer neuroscience is to improve outcomes for people with cancer. Understanding neural–tumor interactions is already providing opportunities to repurpose neuromodulatory drugs for cancer. Findings presented at the Symposium show that the SNS can be effectively targeted in cancer patients, with early phase randomized clinical trials in breast cancer and melanoma providing evidence that β-blockade of SNS signaling downregulates biomarkers of tumor cell invasion and shapes the immune response in tumors (Hiller et al. 2020; Gandhi et al. 2021). To ensure preclinical findings can be translated, it is essential that experiments are designed to consider both disease stage and the therapeutic context. This was demonstrated in a recent study of triple-negative breast cancer that found changes in SNS innervation of tumors after treatment with anthracycline chemotherapy, which is a core component of standard treatment regimens for both early and late stage disease (Chang et al. 2023). Many current standard treatments for cancer, including chemotherapy, radiotherapy, and immunotherapy, rely on an effective adaptive immune response, and tight coupling between the nervous system and the immune system suggests that neural signaling may impact treatment response through effects on immune cells as well as direct effects on cancer cells. In addition to understanding the effects of neuromodulatory drugs on tumor pathophysiology, it will be important to design future prospective trials to also understand their effect on the experience of the cancer patient.

Neurons are a highly understudied component of solid tumors. Answering these questions and many others will expand our understanding of how tumors interact with neural circuits in the body and support the development of novel strategies to treat cancer.
